# Establishment and validation of a nomogram model for predicting the specific mortality risk of melanoma in upper limbs based on the SEER database

**DOI:** 10.1038/s41598-024-57541-w

**Published:** 2024-04-26

**Authors:** Mingju Gao, Bingwei Wu, Xinping Bai

**Affiliations:** Department of Plastic Surgery, The Central Hospital of Wuhan, Tongji Medical College, Huazhong University of Science and Technology, No. 26 Shengli Street, Jiang’an District, Wuhan, 430014 Hubei China

**Keywords:** Melanoma of the upper limbs, Nomogram, Specific death, Competing risk model, Cancer, Computational biology and bioinformatics

## Abstract

For patients with upper limb melanoma, the significance of specific death is more important than that of all-cause death, and traditional survival analysis may overestimate the mortality rate of patients. Therefore, the nomogram model for predicting the specific mortality risk of melanoma in the upper limbs was developed. A population with melanoma in the upper limbs, diagnosed from 2010 to 2015, were selected from the National Cancer Institute database of Surveillance, Epidemiology, and End Results (SEER). The independent predictive factors of specific death were confirmed by the competing risk model of one-factor analysis and multi-factor analysis, and the nomogram was constructed according to the independent predictive factors. 17,200 patients with upper limb melanoma were enrolled in the study (training cohort: n = 12,040; validation cohort: n = 5160). Multi-factor analysis of the competing risk model showed that age, marital status, gender, tumor stage, T stage, M stage, regional lymph node surgery information, radiotherapy, chemotherapy, mitotic cell count, ulcer and whether there were multiple primary cancers, were independent factors affecting the specific death of upper limb melanoma patients (P < 0.05). The nomogram has good predictive ability regarding the specific mortality risk of melanoma in the upper limbs, and could be of great help to formulate prognostic treatment strategies and follow-up strategies that are conducive to survival.

## Introduction

Melanoma originates from melanocytes, and is one of the most aggressive malignant tumors. They easily recur or metastasize. According to the characteristics of the pathogenesis, course and prognosis of melanoma, melanoma mainly includes the following four subtypes: Superficial Spreading Melanoma (SSM), Lentigo Maligna Melanoma (LMM), Nodular Melanoma (NM) and Acral Lentiginal Melanoma (ALM)^1^. Among these four subtypes, the incidence of acral melanoma is the highest in Asian countries. Among the white population, acral melanoma accounts for 1–7% of all malignant melanomas, but among Asian people, acral melanoma accounts for more than 50%. The most common primary sites are palms, toes, fingertips and nails^2^. Population based studies have shown that, compared with non-acral melanoma, acral melanoma has a lower survival rate and a worse prognosis^3^. Because melanomas often metastasize through lymph or blood, and lack effective intervention measures, they are usually found in late stages. However, the survival rate of advanced or metastatic melanoma is extremely low. The overall survival period of this kind of rapidly progressive disease is 6–9 months. The 1-year survival rate is 30%—60%, and the 5-year survival rate is only 16%^4^. Therefore, early prediction and diagnosis of acral melanoma is conducive to reducing mortality and improving disease survival rate through long-term effective prognosis and control.

At present, studies on the mortality risk of acral melanoma mainly focus on all-cause death. However, for this kind of disease with good early prognosis, it is found in long-term follow-up that the risk of non-cancer events affecting the survival and prognosis of patients with acral melanoma is also increasing, such as nervous system diseases, heart disease, leukemia, accidents, etc.^5^. However, the current risk research tools often do not have population variables reflecting such other causes of death, which reduces the accuracy of all-cause mortality prediction tools. The traditional survival analysis only focuses on one endpoint event, and if the competing events are not considered, the risk of death may be overestimated. In a practical sense, considering specific death from other causes is more instructive for clinicians and caregivers than all-cause death. Therefore, for patients with melanoma, it is very important to carry out risk stratification for specific death at an early stage. At present, there is a lack of relevant tool to predict the specific death risk of melanoma in the upper limbs. Therefore, the present study analyzed the independent influencing factors of melanoma specific death in the upper limbs based on the competing risk model, and developed a predictive nomogram model for the specific death risk of melanoma in the upper limbs, which could help broaden researchers’ train of thoughts regarding risk prediction and prognosis research for these patients.

## Methods

### Data collection

**Figure 1:**
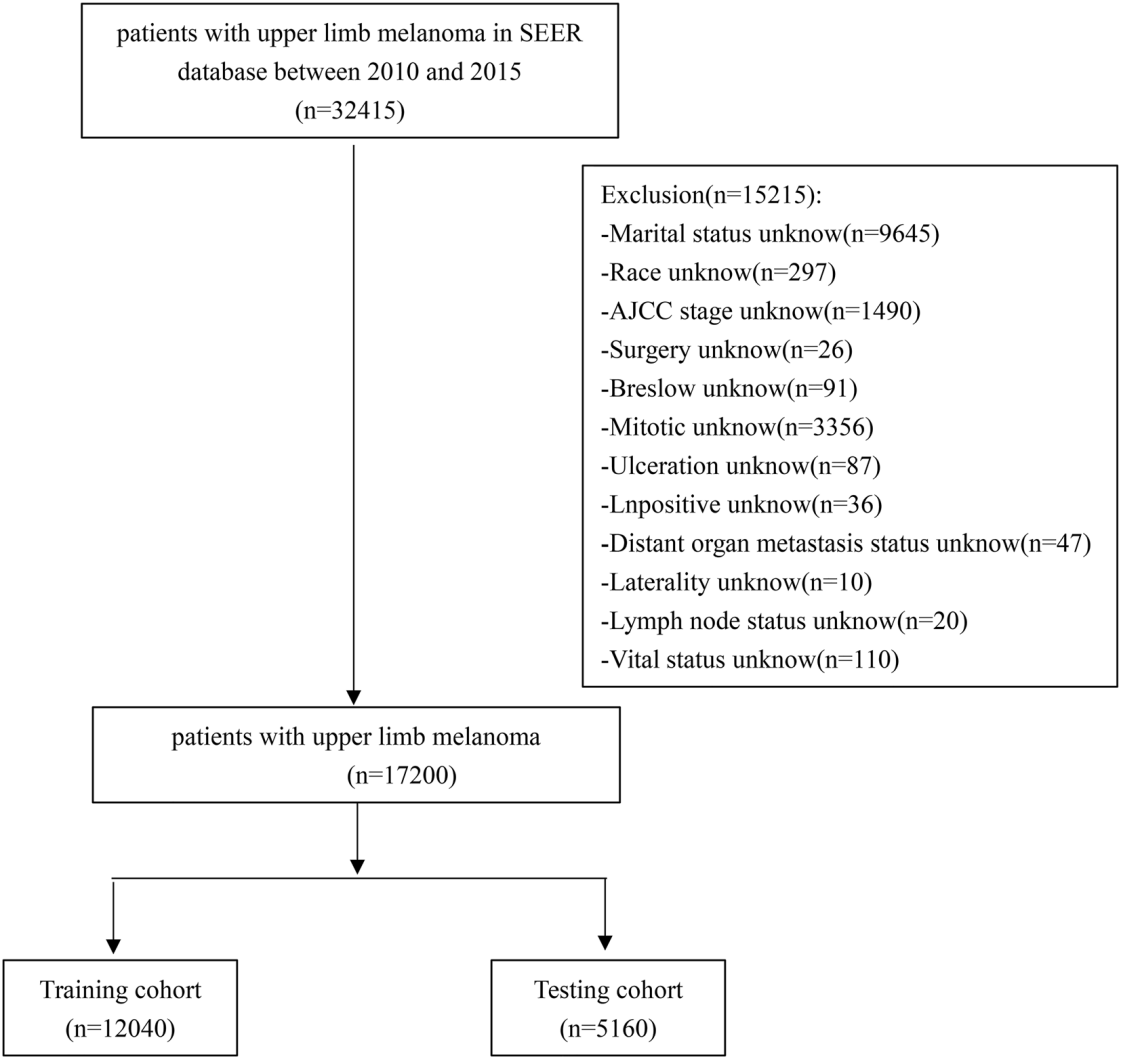
The flowchart of the patients with upper limb melanoma.

The patients who were definitely diagnosed with melanoma by pathology/histology from 2010 to 2015 in the database of Surveillance, Epidemiology, and End Results (SEER) were included as the subjects of this study. Inclusion criteria: (1) The primary site was on the upper limbs; (2) Melanoma was clearly diagnosed by pathology or histology; (3) The diagnosis was made from 2010 to 2015. Patients’ demographic information (age, marital status, gender, race) and clinical information (affected side, tumor stage, T stage, N stage, M stage, operation information of primary tumor, operation information of regional lymph nodes, operation of distant metastasis site, radiotherapy, chemotherapy, Breslow thickness, LDH, number of mitotic cells, ulcer, number of positive lymph nodes, bone metastasis, brain metastasis, liver metastasis, lung metastasis, tumor diameter, number of tumors at diagnosis, whether multiple primary cancers are involved, survival time, cause of death and survival status) were collected. At the same time, these were excluded: (1) survival time < 1 month; (2) Patients with unclear tumor stage, Breslow thickness, affected side, surgical information, mitotic cell number, ulcer and other information at the time of diagnosis; (3) Unknown cause of death (COD). The flow chart of the specific screening process is shown in Fig. 1.

### Construction of competing risk model

In this study, a nomogram of competing risk model was constructed. Therefore, in the data, the death cases caused by other reasons were defined as competing events, the cases that were still alive at the end of the follow-up period were defined as right censoring events, and the cases that passed away from melanoma were defined as interest events. 17,200 samples were randomly divided into a training cohort (70%) and a validation cohort (30%) according to a ratio of 7:3. In the training cohort, single factor analysis and multi-factor analysis of competing risk model were used to screen the independent influencing factors of upper limb melanoma specific death. Variables were discarded due to more than 50% missing values or singular regression problems. According to these independent influencing factors, a risk prediction nomogram model was constructed. The concordance index (c-index) and the areas under the ROC curve (AUC) at different time points were used to evaluate the prediction accuracy of the nomogram, and a larger value indicated a higher prediction accuracy. The calibration curve was used to assess the calibration of the predictive nomogram, and it represents the agreement between the observed and predicted probabilities through 1000 iterations of resampling.

### Statistical methods

R software (Version 4.2.1) and SPSS 26.0 software were used for statistical analysis. Firstly, continuous variables (age, Breslow thickness, number of mitotic cells) were expressed under the form of mean ± standard deviation, and data of the two groups were tested by independent variance T-test. Count data were expressed by case frequency (%), and chi-square test was used for comparison between groups. The patients were randomized into a training cohort, which was used to identify prognostic factors associated with cause-specific death in patients with upper limb melanoma and construct the nomogram, and a validation cohort, which was used to validate the accuracy and effectiveness of the model. Nomograms are common tools for prognostic assessment of tumors, and through nomograms, the complex regression equations are transformed into visual charts, making the results of prediction models more readable and easier for patient assessment. Secondly, traditional survival analysis and competing risk model were used to compare the specific death risk at different time points. After the model was constructed, we calculated the c-index and AUC values in the training and validation cohorts to evaluate the effectiveness and reliability of the prediction model and employed the calibration curve to assess the consistency between the model and the actual situation. P < 0.05 was considered as statistically significant.

## Results

### Clinical characteristics

A total of 17,200 patients were included in this study, and randomly assigned to the training cohort (N = 12,040) and the validation cohort (N = 5160). The average age of patients in the training cohort was 63.41 ± 15.204 years old, 8199 (68.10%) were married, 6482 (53.84%) were male, and 11,933 (99.11%) were of the white race. The average age of patients in the validation cohort was 36.11 ± 15.544 years old; 3542 (68.64%) were married patients, 2795 (54.17%) were male patients, and 5101 (98.86) were patients of white race. 1548 (12.9%) patients died of other causes in the training cohort, and 638 (12.4%) patients died of other causes in the validation cohort. Regarding other variables (Table 1), there was no statistically significant difference between the clinical characteristics of patients in the training cohort and the validation cohort (P > 0.05).

**Table 1:** Clinicopathological characteristics.

	Train (N = 12,040)	Test (N = 5160)	All (N = 17,200)	t/χ^2^ (p)
Age	63.41 ± 15.20	36.11 ± 15.54		1.161 (0.242)
Marriage				
Married	8199 (68.10)	3542 (68.64)	11,741 (68.26)	0.496 (0.481)
Unmarried	3841 (31.90)	1618 (31.36)	5459 (31.74)	
Sex				
Male	6482 (53.84)	2795 (54.17)	9277 (53.94)	0.158 (0.691)
Female	5558 (46.16)	2365 (45.83)	7923 (46.06)	
Race				
White	11,933 (99.11)	5101 (98.86)	17,034 (99.03)	2.452 (0.117)
Other	107 (0.89)	59 (1.14)	166 (0.97)	
Laterality				
Left	6417 (53.30)	2743 (53.16)	9160 (53.26)	0.028 (0.868)
Right	5623 (46.70)	2417 (46.84)	8040 (46.74)	
Stage				
I	9020 (74.92)	3900 (75.58)	12,920 (75.12)	3.969 (0.137)
II	2067 (17.17)	897 (17.38)	2964 (17.23)	
III/IV	953 (7.92)	363 (7.03)	1316 (7.65)	
Tstage				
T1	7565 (62.83)	3240 (62.79)	10,805 (62.82)	1.831 (0.4)
T2	3563 (29.59)	1557 (30.17)	5120 (29.77)	
T3/T4	912 (7.57)	363 (7.03)	1275 (7.41)	
Nstage				
N0	11,138 (92.51)	4808 (93.18)	15,946 (92.71)	2.624 (0.269)
N1	584 (4.85)	233 (4.52)	817 (4.75)	
N2/N3	318 (2.64)	119 (2.31)	437 (2.54)	
Mstage				
M0	11,947 (99.23)	5134 (99.50)	17,081 (99.31)	3.791 (0.052)
M1	93 (0.77)	26 (0.50)	119 (0.69)	
Surgery				
Surgery1	7625 (63.33)	3246 (62.91)	10,871 (63.20)	0.279 (0.598)
Surgery2	4415 (36.67)	1914 (37.09)	6329 (36.80)	
Distant LN				
None	6471 (53.75)	2825 (54.75)	9296 (54.05)	2.108 (0.550)
1 ~ 3	358 (2.97)	161 (3.12)	519 (3.02)	
> 4	467 (3.88)	199 (3.86)	666 (3.87)	
Others	4744 (39.40)	1975 (38.28)	6719 (39.06)	
Surg Oth Reg				
None	11,923 (99.03)	5125 (99.32)	17,048 (99.12)	3.551 (0.059)
Yes	117 (0.97)	35 (0.68)	152 (0.88)	
Radiation				
No	11,922 (99.02)	5113 (99.09)	17,035 (99.04)	0.182 (0.670)
Yes	118 (0.98)	47 (0.91)	165 (0.96)	
Chemotherapy				
No	11,935 (99.13)	5116 (99.15)	17,051 (99.13)	0.016 (0.900)
Yes	105 (0.87)	44 (0.85)	149 (0.87)	
Breslow	1.401 ± 1.793	1.367 ± 1.762		1.149 (0.250)
LDH				
Unhigh	11,656 (96.81)	5004 (96.98)	16,660 (96.86)	0.328 (0.567)
High	384 (3.19)	156 (3.02)	540 (3.14)	
Mitotic	1.94 ± 2.964	1.93 ± 2.99		0.390 (0.697)
Ulceration				
No	10,205 (84.76)	4320 (83.72)	14,525 (84.45)	2.964 (0.085)
Yes	1835 (15.24)	840 (16.28)	2675 (15.55)	
Lnpositive				
No	4795 (39.83)	2011 (38.97)	6806 (39.57)	1.098 (0.295)
Yes	7245 (60.17)	3149 (61.03)	10,394 (60.43)	
DX_bone				
No	12,020 (99.83)	5155 (99.90)	17,175 (99.85)	1.192 (0.275)
Yes	20 (0.17)	5 (0.10)	25 (0.15)	
DX_brain				
No	12,019 (99.83)	5154 (99.88)	17,173 (99.84)	0.779 (0.377)
Yes	21 (0.17)	6 (0.12)	27 (0.16)	
DX_liver				
No	12,024 (99.87)	5156 (99.92)	17,180 (99.88)	0.953 (0.329)
Yes	16 (0.13)	4 (0.08)	20 (0.12)	
DX_lung				
No	11,992 (99.60)	5150 (99.81)	17,142 (99.66)	4.511 (0.034)
Yes	48 (0.40)	10 (0.19)	58 (0.34)	
Tumor size				
< 2	4827 (40.09)	2052 (39.77)	6879 (39.99)	0.456 (0.796)
2–10	745 (6.19)	310 (6.01)	1055 (6.13)	
Other	6468 (53.72)	2798 (54.22)	9266 (53.87)	
Tumor number				
Single	7390 (61.38)	3226 (62.52)	10,616 (61.72)	1.989 (0.158)
Multiple	4650 (38.62)	1934 (37.48)	6584 (38.28)	
First malignant primary				
No	2852 (23.69)	1176 (22.79)	4028 (23.42)	1.621 (0.203)
Yes	9188 (76.31)	3984 (77.21)	13,172 (76.58)	

### Building a competing risk model

**Figure 2:**
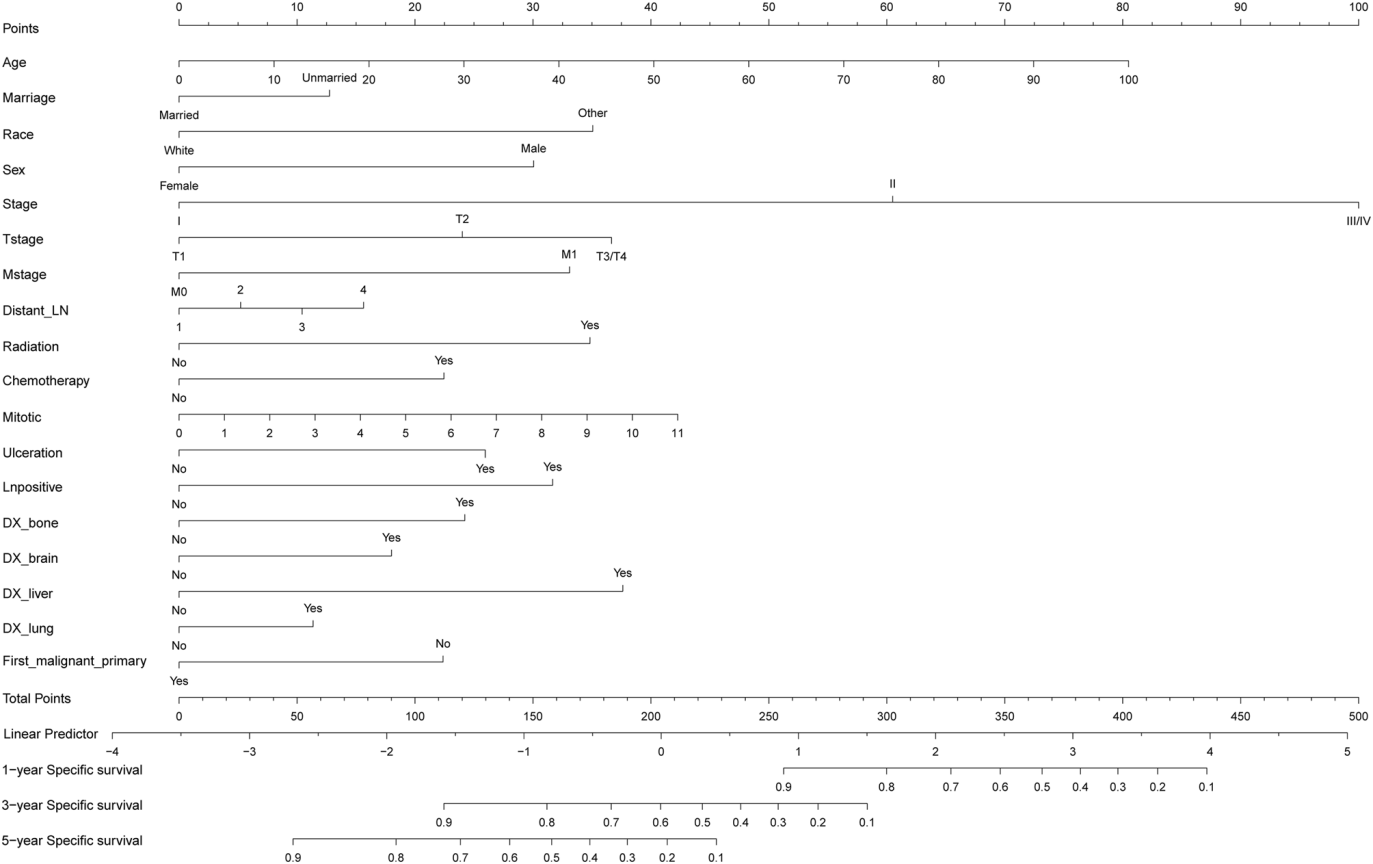
The competitive risk model nomogram of Melanoma of the upper extremity at 1-, 3-, and 5-year.

The results from single factor competing risk model analysis showed that age, marital status, gender, race, tumor stage, T stage, N stage, M stage, primary tumor operation information, regional lymph node operation information, distant metastasis site surgery, radiotherapy, chemotherapy, Breslow thickness, LDH, mitotic cell count, ulcer, number of positive lymph nodes, bone metastasis, brain metastasis, liver metastasis, lung metastasis, tumor diameter, whether multiple primary cancers were involved; all these may cause specific death of melanoma in the upper limbs (Table 2). By substituting these variables into the analysis of the multifactor competing risk model, it was concluded that age, marital status, sex, tumor stage, T stage, M stage, regional lymph node operation information, radiotherapy, chemotherapy, mitotic cell count, ulcer, number of positive lymph nodes, bone metastasis, brain metastasis, liver metastasis, lung metastasis and whether there were multiple primary cancers, were independent influencing factors for the specific death of upper limb melanoma patients (Table 2). Based on these independent influencing factors, a nomogram for predicting the specific mortality risk of melanoma in the upper limbs was constructed (Fig. 2).

**Table 2:** Univariate and multivariate analyses.

	Univariate analysis		Multivariate analysis	
	HR (95%CI)	Z (P)	HR (95%CI)	Z (P)
Age	1.027 (1.021–1.033)	9.169 (< 0.001)	1.019 (1.012–1.025)	5.753 (< 0.001)
Marriage				
Married	Ref	NA	Ref	NA
Unmarried	1.571 (1.349–1.828)	5.823 (< 0.001)	1.259 (1.063–1.492)	2.670 (< 0.05)
Sex				
Male	Ref	NA	Ref	NA
Female	0.484 (0.411–0.571)	− 0.866 (< 0.001)	0.636 (0.532–0.759)	− 4.991 (< 0.001)
Race				
White	Ref	NA	Ref	NA
Other	2.557 (1.560–4.190)	3.726 (< 0.001)	2.005 (1.153–3.486)	2.465 (< 0.05)
Laterality				
Left	Ref	NA	Ref	NA
Right	0.995 (0.857–1.157)	− 0.059 (0.95)	1.116 (0.952–1.310)	1.351 (0.18)
Stage				
I	Ref	NA	Ref	NA
II	6.994 (5.734–8.530)	19.191 (< 0.001)	1.973 (1.382–2.816)	3.743 (< 0.001)
III/IV	21.749 (17.900–26.427)	30.989 (< 0.001)	3.846 (1.707–8.666)	3.250 (< 0.05)
Tstage				
T1	Ref	NA	Ref	NA
T2	5.872 (4.789–7.200)	17.016 (< 0.001)	2.013 (1.406–2.881)	3.825 (< 0.001)
T3/T4	17.415 (13.968–21.710)	25.399 (< 0.001)	2.396 (1.416–4.052)	3.258 (< 0.05)
Nstage				
N0	Ref	NA	Ref	NA
N1	7.716 (6.379–9.333)	21.143 (< 0.001)	0.862 (0.377–1.969)	− 0.353 (0.72)
N2/N3	12.219 (9.872–15.126)	22.997 (< 0.001)	1.041 (0.461–2.354)	0.097 (0.92)
Mstage				
M0	Ref	NA	Ref	NA
M1	26.163 (18.529–36.941)	18.545 (< 0.001)	2.065 (1.011–4.216)	1.991 (< 0.05)
Surgery				
Surgery1	Ref	NA	Ref	NA
Surgery2	1.709 (1.471–1.986)	7.009 (< 0.001)	1.146 (0.967–1.358)	1.576 (0.11)
Distant LN				
None	Ref	NA	Ref	NA
1–3	2.563 (1.732–3.794)	4.705 (< 0.001)	1.664 (0.992–2.791)	1.928 (0.054)
> 4	8.150 (6.397–10.380)	16.989 (< 0.001)	1.936 (1.268–2.954)	3.062 (< 0.05)
Others	2.508 (2.109–2.983)	10.388 (< 0.001)	1.647 (1.132–2.394)	2.612 (< 0.05)
Surg Oth Reg				
None	Ref	NA	Ref	NA
Yes	5.349 (3.595–7.963)	8.266 (< 0.001)	0.765 (0.466–1.256)	− 1.059 (0.29)
Radiation				
No	Ref	NA	Ref	NA
Yes	10.284 (7.514–14.074)	14.557 (< 0.001)	1.337 (0.892–2.006)	1.406 (0.16)
ChemOtherapy				
No	Ref	NA	Ref	NA
Yes	9.401 (6.756–13.082)	13.294 (< 0.001)	1.332 (0.864–2.054)	1.298 (0.19)
Breslow	1.388 (1.360–1.416)	31.219 (< 0.001)	0.994 (0.928–1.065)	− 0.159 (0.87)
LDH				
Unhigh	Ref	NA	Ref	NA
High	2.229 (1.652–3.010)	5.236 (< 0.001)	1.050 (0.722–1.527)	0.257 (0.8)
Mitotic	1.261 (1.242–1.280)	29.779 (< 0.001)	1.062 (1.035–1.089)	4.590 (< 0.001)
Ulceration				
No	Ref	NA	Ref	NA
Yes	6.769 (5.827–7.865)	24.995 (< 0.001)	1.524 (1.241–1.871)	4.022 (< 0.001)
Lnpositive				
No	Ref	NA	Ref	NA
Yes	1.189 (1.018–1.389)	2.188 (< 0.05)	1.834 (1.278–2.632)	3.293 (< 0.001)
DX_bone				
No	Ref	NA	Ref	NA
Yes	42.715 (19.536–93.393)	9.407 (< 0.001)	3.167 (1.587–6.317)	3.271 (< 0.05)
DX_brain				
No	Ref	NA	Ref	NA
Yes	59.745 (29.236–122.092)	11.217 (< 0.001)	4.044 (1.887–8.665)	3.593 (< 0.001)
DX_liver				
No	Ref	NA	Ref	NA
Yes	48.275 (19.546–119.7099)	8.391 (< 0.001)	1.637 (0.602–4.449)	0.966 (0.33)
DX_lung				
No	Ref	NA	Ref	NA
Yes	29.639 (18.632–47.145)	14.311 (< 0.001)	1.927 (0.897–4.138)	1.682 (0.093)
Tumor size				
< 2	Ref	NA	Ref	NA
2–10	2.864 (2.279–3.597)	9.042 (< 0.001)	1.261 (0.956–1.662)	1.641 (0.1)
Other	0.877 (0.745–1.033)	− 1.571 (0.12)	0.935 (0.786–1.111)	− 0.765 (0.44)
Tumor number				
Single	Ref	NA	Ref	NA
Multiple	1.138 (0.978–1.325)	1.669 (0.095)	0.833 (0.649–1.069)	− 1.435 (0.15)
First malignant primary				
No	Ref	NA	Ref	NA
Yes	0.719 (0.611–0.848)	− 3.923 (< 0.001)	0.670 (0.513–0.875)	− 2.937 (< 0.05)

### Validation of the competing risk model

**Figure 3:**
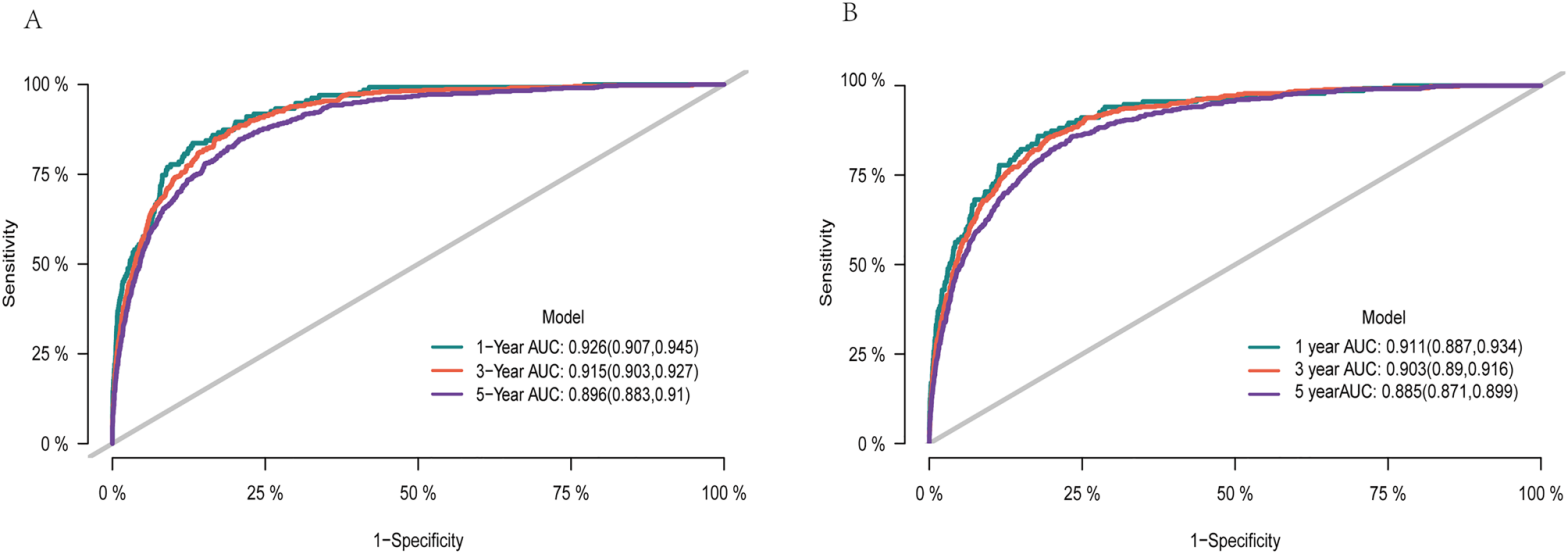
AUC for predicting 1-, 3-, and 5-year in training cohort (**A**) and validation cohort (**B**).

**Figure 4:**
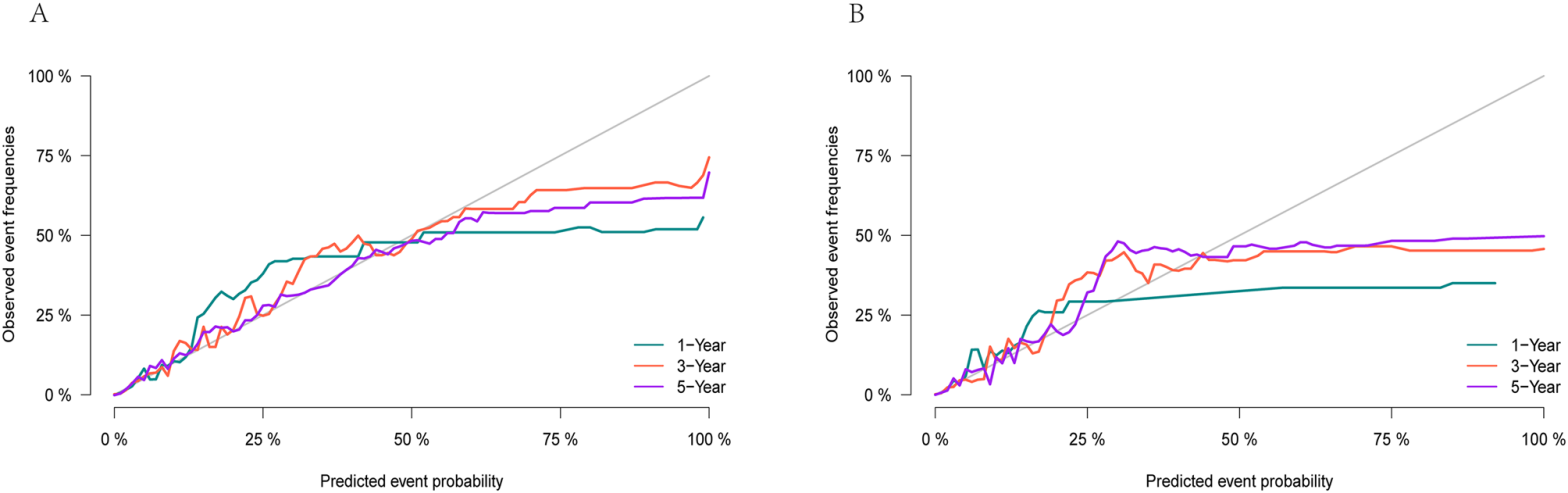
Calibration curve of the nomogram in training cohort (**A**) and validation cohort (**B**).

In this study, the c-index and AUC were used to evaluate the prediction accuracy of the nomogram, and the calibration curve was used to evaluate the calibration of the predictive nomogram. Through the construction of the prediction model of upper limb melanoma specific death, the C-index of the prediction model in the training cohort was 0.894, and the areas under the ROC curve (Fig. 3) of the training cohort at 1, 3 and 5 years was 0.926 (95% CI 0.907–0.945), 0.915 (95% CI 0.903–0.927) and 0.896 (95% CI 0.883–0.910) respectively. At the same time, the calibration curve of the prediction model showed that the predicted risk of the model corresponded well with the actual risk. (Fig. 4). Therefore, the risk prediction model had a quite satisfying prediction effect in the training cohort.

Similarly, the risk prediction model also showed good prediction effect in the validation cohort. The C-index of the prediction model in the validation cohort was 0.875. The AUC of the validation cohort for 1 year, 3 years and 5 years were 0.911 (95% CI 0.887–0.934), 0.903 (95% CI 0.890–0.916) and 0.885 (95% CI 0.871–0.899) respectively (Fig. 3). At the same time, the calibration curve of the prediction model showed that the model had good calibration in the validation cohort (Fig. 4).

### Comparison between traditional survival analysis and competing risk model in mortality estimation

Because the traditional survival analysis only takes into account a single end point event, therefore when the traditional survival analysis estimates the morbidity and mortality, other causes of death are excluded. However, in this study, there is a competitive relationship between the death of patients due to other reasons and melanoma specific death. If the traditional survival analysis is used, it will lead to deviation in the prediction and estimation of the results; overestimating the mortality of melanoma patients, and underestimating the survival possibility of the patients. According to the comparison, at different time points, between the traditional survival analysis and the competing risk model in estimating the cumulative mortality of melanoma in the upper limbs in this study (Table 3), the mortality estimated by the traditional survival analysis and the competing risk model were significantly different. The mortality estimated by the traditional survival analysis was higher than that of the competing risk model.

**Table 3:** Cumulative specific mortality at different time points in survival analysis and competing hazards models.

Time points (month)	Survival analysis mortality	Competing risk model	
		Specific mortality	Other causes mortality
12	1.261	1.100	2.111
24	3.106	2.707	4.543
36	4.560	3.973	7.000
48	5.480	4.767	9.252
60	6.157	5.342	11.691
72	6.855	5.926	13.680
84	7.526	6.478	15.984
102	8.403	7.186	19.417

## Discussion

Based on the SEER database, the study found that the number of deaths from other causes in upper limb melanoma patients accounted for 12.7% of the total number. In order to avoid prediction bias caused by deletion, the study object could not exclude these patients who died from other causes. Therefore, it was impossible to build a specific death risk prediction based on traditional survival analysis. For this reason, this study used the competing risk model to construct the risk prediction nomogram of upper limb melanoma. Through internal and external validation, it was concluded that the nomogram had very ideal risk assessment accuracy (the C-index in the training cohort was 0.894, and the C-index in the validation cohort was 0.875). At the same time, the study found that there was considerable difference between the mortality estimated by the traditional survival analysis and the competing risk model, which also confirmed Lacny et al.^6^ conclusion that the traditional survival analysis overestimates the cumulative morbidity in the clinical field.

At present, predictive nomograms of melanoma have been widely established. Jun Tian et al. used the Kaplan–Meier method of survival analysis and COX proportional-hazards regression model to build a nomogram, based on three long non-coding RNAs (lncRNAs), to predict the overall survival rate of skin melanoma. The AUC of the validation cohort for 3, 5, and 10 years were 0.717, 0.724, and 0.633, respectively^7^. Deng et al. used the traditional survival analysis method, by combining autophagy related gene characteristics and clinical parameters, to construct and validate the prognostic nomogram (C-index, 0.717) of melanoma patients. The AUC values for 3 and 5 years were 0.790 and 0.760^8^. Sandra L. Wong et al. developed a nomogram to predict the positive probability of sentinel lymph nodes in melanoma patients (C-index, 0.694)^9^. Verver et al., based on Cox regression analysis, constructed and validated a nomogram of recurrence of sentinel node negative patients and melanoma specific mortality, with a c-index of 0.76^10^. Most studies use the results of traditional survival analysis to construct all-cause mortality nomograms. Although they can be used to predict the cumulative survival rate of melanoma, they are not accurate enough. Li et al., based on the competing risk model, developed a nomogram on cancer specific mortality of patients with metastatic cutaneous melanoma. The results showed that the AUC values at 6 months, 12 months and 18 months in the training cohort were 0.706, 0.700 and 0.706 respectively, and 0.702, 0.670 and 0.656 in the validation cohort respectively^11^. More accurate prediction models are needed for the competing survival risk of patients with melanoma in the upper limbs. Our study, based on the competing risk model, constructed the risk prediction nomogram of upper limb melanoma specific death. The C-index in the training cohort was 0.894, and the AUC in the training cohort for 1, 3, and 5 years was 0.926, 0.915, and 0.896 respectively. The results showed that, regarding the construction of melanoma risk prediction, the competing risk model was more effective.

This study found that the death risk of melanoma increases with age, which is consistent with some already published research results. Age is a key factor in the occurrence of all cancers. With age, the risk of gene mutation that may cause cancer increases^12^. Older patients with melanoma are more likely to have thicker tumors, higher mitotic rates, and are more prone to ulceration^5^. Marital status is also one of the predictors of the nomogram in this study. The cumulative survival rate of unmarried melanoma patients was lower than that of married patients. Previous studies have shown that compared with single status patients (single, widowed or divorced), married patients (including men and women) had a 35% lower risk of death after melanoma diagnosis^13^. This may be because married patients have better social support and living environment^11^. At the same time, we also found that women have a higher cumulative survival rate, which is consistent with other research results^12^. The survival rate of women in the early stages of melanoma (stage I and II and stage III) is higher than that of men^14^. The incidence of acral melanoma is related to race; the incidence rate in colored people is significantly higher than that in Caucasians^15^. TNM staging is one of the important independent factors for melanoma risk prediction, and is an internationally recognized prognostic factor of cancer. It is the most commonly used staging and prognostic assessment tool in clinical practice. AJCC staging provides the most important initial classification for melanoma patients^16^, which is of great significance for the prediction of melanoma specific death risk^10^. A large number of studies^12,17,18^ emphasized the importance of mitotic rate for the prognosis of melanoma. Statistically, except for thickness, the mitotic rate is the most effective survival predictor. One or more mitotic rates per square meter are related to the significant reduction of survival rate^17^. Our research also confirmed this point. At the same time, we also realized that ulcer is an important independent influencing factor of melanoma specific death^5^. The presence of ulcer may reflect the relatively rapid growth of melanoma. Previous research results showed that in the presence of ulcer, the five-year survival rate of stage I–II melanoma decreased from 80 to 55%, and that of stage III melanoma decreased from 53 to 12%^19^. When bone metastasis appear in melanoma, the survival rate of patients will be significantly reduced. The research results of Melissa A Wilson et al. proved this point. Clinically, melanoma will be difficult to treat when it spreads to the skeletal system^20^. Melanoma brain metastasis (MBM) is very common, and is related to a particularly poor prognosis. They directly lead to 60–70% of melanoma patients' death^21^. Our research also proved that brain metastasis of melanoma can seriously affect the survival rate of patients. The univariate competing risk model suggested that chemotherapy was a risk factor for upper limb melanoma. Previous studies have shown that metastatic melanoma has a very low response rate to single-agent chemotherapy, and multiple chemotherapy methods improve the response rate but do not significantly improve the overall survival (PMID: 12407508, PMID: 12407507). For advanced malignant melanomas, radiotherapy and chemotherapy are still relatively ideal treatment methods^22^, which can reduce the recurrence risk of patients to a certain extent, and prolong their survival period^23^. More studies are needed to clarify whether chemotherapy alone can improve the survival rate in melanoma.

Melanoma is a cancer with good prognosis at early stages. The 10-year metastasis free survival rate is 91.8–99.5%^24^. Therefore, in the long-term follow-up process, there may be deaths due to other causes, such as heart disease, nervous system disease, leukemia, etc. In our study, 69.5% of upper limb melanoma patients died due to other reasons. When predicting the death risk of upper limb melanoma, these competing influencing outcomes should be considered. This study explored disease mortality based on traditional survival analysis and competing risk model for different end points. The results showed that the mortality estimated by traditional survival analysis was higher than that of the competing risk model. At the same time, in the competing risk model, the mortality caused by other reasons was higher than the specific mortality. Therefore, in this study, the traditional survival analysis may have overestimated the mortality of melanoma in the upper limbs due to the deletion of outcome variables. This is consistent with the results of other diseases that used different analysis methods to estimate mortality^5,25^. Therefore, when using the competing risk model to predict the mortality risk of melanoma in upper limbs, this study obtained good evaluation efficacy.

The nomogram of upper limb melanoma specific death developed in this study has good predictive effect. Compared with the traditional survival analysis and prediction tools, it also has better predictability. However, as this study was based on one same database for data validation, it did not use real clinical external data for prospective validation. This kind of studies will continue to be completed in follow-up work.

## Conclusion

Under the circumstance that competing events accounted for a large proportion, competing risk model seems to be a more reasonable tool for studying diseases’ specific death. The risk prediction nomogram constructed in this study, based on this method, had better accuracy and good prediction capacity for predicting the specific death risk of melanoma in upper limbs. This model can provide a reference basis for melanoma risk stratification, and can also help to formulate prognostic treatment strategies and follow-up strategies conducive to survival.

## Data Availability

The data that support the findings of this study are available from the corresponding author upon reasonable request.
